# Measuring Problematic Facebook Use among Adolescents and Young Adults with the Bergen Facebook Addiction Scale: A Psychometric Analysis by Applying Item Response Theory

**DOI:** 10.3390/ijerph18062979

**Published:** 2021-03-14

**Authors:** Caterina Primi, Giulia Fioravanti, Silvia Casale, Maria Anna Donati

**Affiliations:** 1 Department of Neuroscience, Psychology, Drug, and Child’s Health (NEUROFARBA), Section of Psychology, University of Florence, 50100 Florence, Italy; caterina.primi@unifi.it (C.P.); mariaanna.donati@unifi.it (M.A.D.); 2 Department of Health Sciences, Psychology Unit, University of Florence, 50100 Florence, Italy; silvia.casale@unifi.it

**Keywords:** Facebook addiction, problematic Facebook use, adolescents, BFAS, IRT, gender/age invariance, DIF

## Abstract

The Bergen Facebook Addiction Scale (BFAS) is widely used, but psychometric evidence by applying Item Response Theory (IRT) is lacking. Considering the advantages of this psychometric approach, the aim of study was to investigate the psychometric properties of the Italian version of the BFAS among adolescents and young adults. Participants were 1134 (50% males, Mean age = 20.7, SD = 3.5, range = 14–33 years) Italian high school students and undergraduates. The unidimensionality of the scale was confirmed (*χ^2^*/df = 2.8, *CFI* = 0.99, *TLI* = 0.98, and *RMSEA* = 0.04 [*C.I.* = 0.02–0.06]) and IRT analysis showed that the scale assesses medium and high levels of the trait, and that it is useful in order to discriminate different levels of Problematic Facebook use (PFU) within this range of trait, in which the scale is sufficiently informative. The relationships of BFAS *θ* scores with theoretically related constructs provided support to the validity of the scale. In accordance with previous studies, BFAS scores were positively correlated with Problematic Internet use and problematic Social Network use, negatively correlated with self-esteem, and positively related to loneliness. The Differential Item Functioning (DIF) analysis showed that BFAS is invariant across gender, and only one item had uniform and small-in-size DIF. Additionally, we tested age invariance. Since only 17% of the BFAS items were non-invariant, we determined that the BFAS exhibited minor non-invariance as a whole. An analysis of the adequacy of the polythetic and monothetic criteria to define the range of the trait indicative of problematic use was also conducted. Overall, this study offers evidence that BFAS is a valuable and useful scale for measuring high levels of PFU among Italian adolescents and young adults.

## 1. Introduction

The use of social networking sites (SNSs) has rapidly increased over the last few years. Although conflicting positions exist, some authors [1] have suggested that SNS use may have the potential to become addictive as some users report cognitive and behavioral symptoms similar to those experienced by those suffering from recognized addictions (e.g., gambling disorders). Mood modification, salience, withdrawal symptoms, and conflict seem to exist in people who use SNSs excessively [2,3,4]. Andreassen and Pallesen describe problematic SNSs use as “being overly concerned about SNSs, to be driven by a strong motivation to log on to or use SNSs, and to devote so much time and effort to SNSs that it impairs other social activities, studies/job, interpersonal relationships, and/or psychological health and well-being” [5] (p. 4054). Over the past few years, research in the field of problematic SNSs use (PSNSs) has largely shifted from the study of generalized PSNSs use to the use of Facebook in particular (for a meta-analysis, see [6]). In 2019, Facebook was the most popular SNS with 2.41 billion monthly active users [7]. Facebook has long the highest number of active members, with a 9% increase in daily active users year-on-year [8]. Five new profiles are created every second and 510,000 comments are posted, 293,000 statuses updated, and 136,000 photos uploaded every minute, whilst the average daily time spent on the site is 20 min [8].

Problematic Facebook use has been defined as Facebook use that creates problems in users’ life, such as psychological, emotional, social, school, or work difficulties [9]. Different conceptualizations coexist in the literature [6]. For example, “Facebook addiction” is defined either by the six criteria of addiction (i.e., salience, mood modification, tolerance, withdrawal, conflict, and relapse) or by similar factors based on the definition of gambling addiction (e.g., withdrawal, interpersonal problems due to Facebook use, time management, and performance problems) [10,11]. The term “Facebook intrusion” [12] has been used to define an excessive involvement in Facebook that disrupts everyday activities and duties, manifesting itself in the compulsive use of the site and in the neglect of social life. The term “problematic Facebook use” (PFU) includes both addictive-like symptoms and specific features such as the preference for online social interaction as a means of mood regulation [9,13]. Although different, all these conceptualizations speculate that PFU should be considered a nonsubstance-addiction, i.e., a behavioral disorder (also called behavioral addiction) not related to any substance of abuse that shares some features with substance-induced addiction [14].

The first meta-analysis that summarized research on the association between PFU and psychological wellbeing in adolescence and early adulthood showed that PFU is positively correlated with psychological distress, including anxiety and depression, and negatively correlated with well-being (including life satisfaction and other indicators of subjective wellbeing) [9]. Moreover, a small gender effect favoring women was found [9]. An explanation for this gender difference may lie in women’s preference for online social interaction [15] that puts them at greater risk of presenting more Facebook addiction symptoms [16] than men who have been shown to be more involved in other online activities such as gaming [17]. PFU was positively associated with time spent online and Internet addiction. The correlation between PFU and Internet addiction suggested that the former could be considered a subtype of the latter but also that they are not fully overlapping phenomena, suggesting the importance of studying PFU as a unique phenomenon [9]. 

Studies from numerous countries report different PFU prevalence rates—between 2% and 10% among adolescents and young adults worldwide [9], mainly due to the absence of a clear and effective definition of this construct and the use of different evaluation instruments (i.e., the Facebook Addiction Scale, the Facebook Intrusion Questionnaire, the Bergen Facebook Addiction Scale). They were often developed as ad-hoc measures or adapted from items originally used to assess other behavioral addictions (for a review see [18]). For example, the Facebook Addiction Scale [19,20,21] is a modified version of Young’s Internet Addiction Test [22], whereas the Facebook Intrusion Questionnaire [12] is based on the Mobile Phone Involvement Questionnaire [23]. The most extensively used measure is the Bergen Facebook Addiction Scale (BFAS) [10], based on the six core components of behavioral addiction distinguished by Griffiths [11]: Salience, mood modification, tolerance, withdrawal symptoms, conflict, and relapse. At first, three items for each component were selected. The wording of the items was analogous to that used in the diagnostic criteria for pathological gambling [24] and in the Game Addiction Scale [25]. Of the three items within each of the six core components, the one with the highest item-total correlation was held. Consequently, only six items composed the final version. Each item refers to the past 12 months and is answered on a 5-point Likert scale ranging from 1 (very rarely) to 5 (very often). 

BFAS has satisfactory psychometric properties with regard to internal consistency (Cronbach’s alpha = 0.83), factor structure (unidimensional), and reliability (*r* = 0.82 for re-administration after three weeks), as well as in relation to content and convergent and discriminative validity. Specifically, the BFAS scores were positively correlated with Facebook measures of addictive tendencies, attitudes, and online sociability. Factor loading invariance across gender was also demonstrated [10]. The authors suggested that the BFAS can be used in epidemiological as well as clinical settings. Cut-off scores for a classification of Problematic Facebook users were not established for the BFAS. However, to determine the cut-off score of the scale, the authors proposed using either polythetic e.g., responding 3 or above in the response scale on at least four of the six items, thus having at least a summed total score of 12, or monothetic scheme, e.g., responding 3 or above in the response scale on all six items, thus having at least a summed total score of 18 [10]. 

The BFAS psychometric properties have been assessed in different international studies, including Poland [26], Portugal [27,28,29], Brazil [30], Egypt [31], Tunisia [32], Peru [33], Pakistan [34,35], Bangladesh [36], Thailand [37], Turkey [38], Iran [39], and Italy [40]. Results demonstrated that satisfactory psychometric properties were found, including reliability and convergent validity. A one-factor solution emerged from all the studies. Gender invariance was tested and confirmed at configural, metric, and scalar level in two studies [33,34]. The majority of the psychometric studies were performed among young adults (i.e., university students) and three studies were conducted on adolescents (i.e., high school students). Only one study [33] tested age invariance providing evidence for configural and metric invariance of the BFAS scale across the age groups (participants aged under 20 years vs. participants aged over 20 years), but not for scalar invariance. 

Regarding convergent validity, the BFAS scores were found to be positively associated with Problematic Internet Use and Internet Addiction [27,28,29,30], fear of missing out [33], social anxiety, loneliness, and depression [26,29,32]. Extraversion, narcissism, and low self-efficacy were also found to be associated with BFAS scores [26].

Since PFU diffused to various cultural contexts, it seems important to confirm the properties of the scale in different cultures. In this regard, recently, the Italian BFAS psychometric properties have been explored [40]. A confirmatory factor analysis confirmed the construct validity demonstrating that it assesses a unidimensional construct. The validity was investigated through the correlation between the total score of the BFAS with a series of variables, which have been associated with PFU and generalized problematic SNSs use (e.g., frequency of SNS use, problematic Smartphone use, anxiety, depression). Additionally, the convergent validity was analyzed measuring the correlation with the Italian version of the Bergen Social Media Addiction Scale -BSMAS [41] as both scales concern problematic SNSs use (BFAS more specific and BSMAS more general). Finally, the reliability was assessed using several indices. Both Cronbach alpha coefficient and McDonald’s ordinal Omega index [42] were excellent, at α = 0.94 and ω = 0.95, respectively. 

However, the psychometric study of the scale has been carried out by applying classical test theory (CTT), while there are no IRT contributions.

### Aims of the Study

Starting from this premise, in the current study, we aimed to extend the investigation of the psychometric properties of the Italian version of the Bergen Facebook Addiction Scale (BFAS) [10] by adopting the Item Response Theory (IRT) approach. Indeed, the IRT modes offer important advantages to understand the potential of a given instrument. IRT assumes that the probability of an item response depends on the latent trait of the respondents, called *θ*, and the properties of items on a test (i.e., item parameters). Along with the discriminative power (*a*), IRT allows us to analyze item location, i.e., the “severity” of the symptom described by the item (*b* parameters). With IRT analyses we can evaluate how well an item performs in measuring the underlying construct, the level of the construct targeted by the item, and the appropriateness of the response categories [43]. 

Concerning reliability, IRT provides the test information function (TIF) to evaluate the precision of the test at different levels of the measured construct, instead of providing a single value (e.g., Cronbach’s α) [44,45]. In detail, the TIF provides information on the accuracy of the test at estimating a trait along the whole range of trait scores: The more information the test provides at a particular trait level, the smaller the error associated with *θ* estimation, and the higher the local reliability. Another advantage of IRT is latent trait estimation. In IRT, the latent trait scores (*θ* values) can be estimated by using the model parameter estimates by searching for values that maximize the likelihood of observed patterns of responses to all the items in the test [46].

In detail, we used IRT trait estimates for the BFAS to analyze the validity of the scale. Applied research showed that the IRT summed-score approach is a valid method than can be applied to various research purposes [47,48], as in the evaluation tests validity [49].

Additionally, the IRT allows for the assessment of differential item functioning (DIF) [43], an efficient method for analyzing a test’s measurement invariance [50]. DIF analysis tests the performance of items examining whether or not the likelihood of endorsing each item is equal across subgroups of respondents that are matched on the measured trait. For example, a randomly selected man with a certain level of *θ* and a randomly selected woman with the same level of *θ*, should have the same likelihood of endorsing a certain response option for each item on a test. 

In this study, we analyzed gender and age DIF. Gender invariance was already tested in previous studies. However, it has not been yet investigated with the Italian version. Age invariance was investigated only in one previous study [33]. In this study, we tried to fill this gap; for this reason, participants were of a wide range of age in order to investigate invariance across age. Assessing measurement invariance is useful for determining if the trait scores between groups are comparable and have the same meaning across the groups [51]. Indeed, until it is determined that a measure assesses the same trait across separate groups, comparisons among these groups on the measure have uncertain meaning [52]. Invariance also allows us to better investigate the impact of gender and age differences on the study of PFU among adolescents and young adults. 

The first step was to investigate the scale’s unidimensionality (e.g., [10,26,28]). We then analyzed severity and discrimination of the items, as well as the accuracy of the scale along the continuum of the trait with the TIF. Next, we measured gender and age invariance through DIF analyses, after which we investigated gender and age differences. Then, IRT latent scores were computed to analyze the validity of the Italian version of the BFAS. In accordance with previous validation studies (e.g., [26]), we expected that BFAS scores would be positively correlated with Problematic Internet use, generalized Problematic SNS use, and loneliness, and negatively correlated with self-esteem.

Finally, through a joint analysis of the TIF and correspondence between IRT latent scores and traditional summed scores, we defined a range of BFAS scores that could be adequately considered as indicative of a pathological behavior. Indeed, the proposed cut-off scores [10] consider items as equivalent in terms of severity, as a response equal or higher than 3 on the item response scale was counted as 1—in both of the cut-off criteria—without considering the specific weight of the symptom described by the different items. Instead, *θ* values are weighted both on the parameters of each item endorsed and on the specific response category selected for each item. 

In conclusion, the aims of this work were to confirm and extend analyses of the psychometric properties of the Italian version of the BFAS, by applying IRT. 

## 2. Materials and Methods

### 2.1. Participants 

Participants were 1134 (50% males, *mean age* = 20.7, *SD* = 3.5, range = 14–33 years). Sixty-five percent of the participants (*n* = 736) attended university. University participants were recruited among students attending the School of Psychology at the University of Florence (Italy). After providing informed consent, students were asked to complete the questionnaire pack in class. Participation was voluntary, anonymous, and had no impact on the students’ academic record. Thirty-five percent of the participants (*n* = 398) attended different Italian high schools (45% Lyceum, 46% vocational institutes, and 9% Technical colleges). The high school sample was recruited in the urban centers of Florence and Pisa, Italy. Study protocol was approved by each school’s institutional review board. The students received an information sheet, which guaranteed them that the data acquired would be treated confidentially and anonymously, and they were asked to give written informed consent. Parents of minors were asked to provide consent on behalf of their children.

### 2.2. Measures and Procedure

The Italian version [40] of the BFAS [10] was used. It comprises six items (e.g., “How often during the last year did you use Facebook in order to forget about personal problems?”) scored between 1 (very rarely) and 5 (very often) with higher scores denoting higher levels of PFU. The Italian BFAS showed good internal consistency in this sample (α = 0.94).

The Italian version [53] of the Generalized Problematic Internet Use Scale 2 (GPIUS2) [13] was used to assess the types of cognitions, behaviors, and outcomes that arise because of the unique communicative context of the Internet. In the present study, participants were asked to refer to their use of SNSs. The GPIUS2 contains 15 items rated on an 8-point Likert scale (from “definitely disagree” to “definitely agree”). Participants’ scores on the 15 items can summed to produce an overall GPIU2 score. Cronbach’s alpha in the current study was 0.89.

The Italian version [54] of the Internet Addiction Test (IAT) [22] was used. The IAT contains 20 items on a scale ranging from 1 (never) to 5 (always). A sample item is, ‘‘How often do you find that you stay online longer than you intended?’’ In the current study, the IAT shows good internal consistency (α = 0.87).

Perceived loneliness was assessed by means of the Italian Loneliness Scale (ILS) [55], a 20-item self-report scale; 18 items were adapted from the University of California Loneliness Scale [56] and the Dutch De Jong-Gierveld Loneliness Scale [57] whereas two items were created ad hoc for the Italian scale. The ILS encompassed three subscales: Emotional loneliness subscale includes six items on emotional abandonment and missing companionship; social loneliness subscale comprises five items that assess feelings of sociability and of having meaningful relationships; general loneliness subscale was composed by seven items (i.e., the items that Oshagan and Allen [58] found to be the most reliable ones from their analysis of the UCLA 10-item version) that measure feelings of isolation. Each item was answered on a scale ranging from 1 (never) to 4 (always). The ILS is a valid and reliable instrument for measuring perceived loneliness among the Italian population [58]. In the current study, the ILS shows excellent internal consistency (α = 0.92).

Self-esteem was measured by the Italian version [59] of the 10-item Rosenberg Self-Esteem Scale (RSES) [60]. This scale has good internal consistency (α = 0.84). Each item was scored on a 5-point Likert scale. A sample item is ‘‘On the whole, I am satisfied with myself.’’ In the current study, the RSES shows good internal consistency (α = 0.81).

All participants completed the BFAS and then the GPIUS. A subsample of the high school students (*n* = 184) also fulfilled the IAT, the ILS, and the RSE, in this order, to examine criterion validity. Both high school and university students completed the scale individually in class during the school time and under the supervision of a trained research assistant. 

### 2.3. Statistical Analysis

Preliminarily, the one-factor structure of the BFAS was tested to assure that the item parameter estimates properly reflect the latent trait and are not biased by additional dimensions. As an important preliminary step, we examined the assumptions of the scale’s unidimensionality through a confirmatory factor analysis in the total sample, employing the Mean-Adjusted Maximum Likelihood (MLM) estimator (Mplus software) [61]. This estimator provides the Satorra–Bentler Scaled chi-square (SB*χ^2^*) [62], an adjusted and robust measure of fit for non-normal sample data, more accurate than the ordinary chi-square statistic [63]. To verify the models’ fit, the ratio of chi-square to its degrees of freedom (*χ^2^*/df); the comparative fit index (CFI) [64]; the Tucker–Lewis index (TLI) [65]; and the root mean square error of approximation (RMSEA) [66] were taken into account. In the case of *χ^2^*/df, values below or equal to two are considered good, while values between two and three are considered acceptable [67]. For the TLI and CFI indices, values above 0.90 are indicative of acceptable fit, while values above 0.95 are indicative of excellent fit [67]. The RMSEA value is considered acceptable when it is below 0.08 and good when it is below 0.05 [68]. 

IRT analyses were conducted using IRTPRO software [69] and, according to the response format, Samejima’s [70] graded response model (GRM), the most commonly two-parameter (2PL) logistic model used IRT model in clinical assessment (for a review see [71]), was applied. First, we used the *χ^2^*LD statistic [72] to test the presence of local dependence (LD), i.e., an excess of covariation among item responses that is not accounted for by a unidimensional IRT model. Values of 10 or greater suggest the presence of a multifactorial structure. Then, the GRM analyses were conducted. In this model, the probability that a response should be in category *k* or higher for each value of trait (*θ*) is estimated. The curve that relates the probability of an item response to the underling trait measured by the item set is the Response Characteristic Curve (RCC). This curve is characterized by an average discrimination parameter across response categories (*a*) and location (also called threshold, or severity) parameters (*bi*). Thus, the GRM will estimate only one discrimination parameter, while the number of threshold parameters per item will correspond to the number of response options minus 1. IRT model fit is evaluated using M_2_ statistic and the associated RMSEA value. As M_2_ statistic is generally unrealistic because of some error in any strong parametric model [73], the RMSEA provides a better metric for model error [74]. Values of RMSEA of 0.05 or less indicate good fit. Item parameters were estimated by employing the marginal maximum likelihood estimation method with the expectation–maximization algorithm [75] implemented in IRTPRO.

The item characteristics estimated in the 2PL model are enable an evaluation of how well an item performs in measuring the underlying construct, the level of the construct targeted by the item, and the appropriateness of the response categories [76]. The discrimination parameter indicates the ability of an item to discriminate among people holding different levels of the underlying trait. Following Baker and Kim [77], discrimination parameter values comprised from 0.01 to 0.34 are interpreted as very low, from 0.35 to 0.64 are considered low, from 0.65 to 1.34 are interpreted as moderate, from 1.35 to 1.69 are high, and 1.70 or higher values are interpreted as very high. In clinical assessment, values equal to 1 or greater are considered substantial (e.g., [78]). Additionally, through the TIF, IRT makes it possible to assess the measurement precision of the test, at different levels of the measured construct [43,44]. The information (I) is the expected value of the inverse of the error variances for each estimated value of theta [I(*θ*) ≈ 1/SE^2^(*θ*)]. The associated reliability is 1 minus the inverse of the information the test provides [*r* = 1(1/I)]. This means that the more information the test provides at a particular trait level, the smaller the error associated with trait estimation. Graphically, the TIF shows how well the construct is measured at different levels of the underlying construct continuum. 

To study the validity of the scale, first we calculated IRT estimate scores of *θ* values, which allow us to estimate the trait level of each respondent simultaneously with the item parameters. IRT estimate scores were computed with the EAP estimation method [79], which is an excellent computational option for unidimensional scales [80]. Finally, Pearson product-moment correlations between the BFAS *θ* values and the GPIUS, IAT, and ILS total scores were computed. 

Analyses of DIF across genders and ages were then performed by applying the IRT likelihood ratio test approach (IRTLR) [81] with IRTPRO [69]. Through this procedure, differences in log-likelihoods (distributed as chi-square) associated with nested models were compared. Two types of DIF can be detected in the GRM model: Uniform DIF (for the location parameters) and nonuniform DIF (for the discrimination parameter). As multiple tests were performed, the level of significance of 0.05 was adjusted by Bonferroni correction to 0.004 (0.05/12). To determine if the detected DIF is meaningful [82], we also analyzed the magnitude of the DIF. Following guidelines established by Kim et al. [82], we calculated non-compensatory DIF (NCDIF) [83], which focuses on item-level expected scores—that is, the sum (over categories) of the probability of response in category *k*, weighted by the category score (i.e., the ordinal code for the category; [84]). Once an item with significant DIF is detected, the average of the squared difference between expected item scores for individuals as a member of the focal group and as a member of the reference group is calculated [85,86,87]. According to Raju [88] 0.096 is the highest cut-off value recommended for polytomous items with five response options. 

Finally, to give indications to improve the scoring system of the BFAS, we looked at the TIF by searching for the region of the trait characterized by the highest information, and then we analyzed the correspondence between IRT latent scores and traditional summed scores, in order to define a range of the BFAS traditional summed scores that may be considered as indicative of a pathological behavior in relation to the information capacity of the BFAS.

## 3. Results

Preliminarily, item distributions and descriptives were investigated to assess normality. Skewness and Kurtosis indices of the items revealed that the departures from normality were not acceptable [89] (Table 1). 

Then, the original factor structure was tested by CFA employing the MLM estimator (Mplus software) [61]. The fit indices of the unidimensional model were not acceptable (*χ^2^*/*df* = 9.5; *CFI* = 0.95, *TLI* = 0.91, and *RMSEA* = 0.09 [95% *CI* = 0.07–0.10]). Modification indices (MIs) suggested adding error covariance between item 1 (spending a lot of time thinking about Facebook or planned use of Facebook) and item 2 (feeling the urge to use Facebook more and more). The modified model showed a good fit (*χ^2^*/*df* = 2.8, *CFI* = 0.99, *TLI* = 0.98, and *RMSEA* = 0.04 [*C.I.* = 0.02–0.06]). All factor loadings were significant (*p* < 0.001), ranging from 0.58 to 0.87 (Table 1). None of the LD statistics were greater than 10, attesting that there was not an excess of covariation among item responses when *θ* was held constant.

After having verified the scale’s unidimensionality, unidimensional IRT analyses were conducted by applying Samejima’s [70] GRM model. The fit statistics indicated an adequate fit (*M*_2_ = 517.41, *df* = 234, *p* < 0.0001; *RMSEA* = 0.03). Concerning the item parameters estimates, discrimination parameter values were high for Item 4 and Item 6, and very high for Item 1, Item 2, and Item 5. Only Item 3 discrimination value was moderate. Threshold parameters were evenly spaced for all of the items. The item parameters covered from about 0.50 *SDs* above the mean to about 2 *SD*s and half above the mean value along the trait continuum. Thus, the item response categories provided an adequate differentiation in measuring the level of the trait around the mean to the high level of the trait (Table 1). 

**Figure 1:**
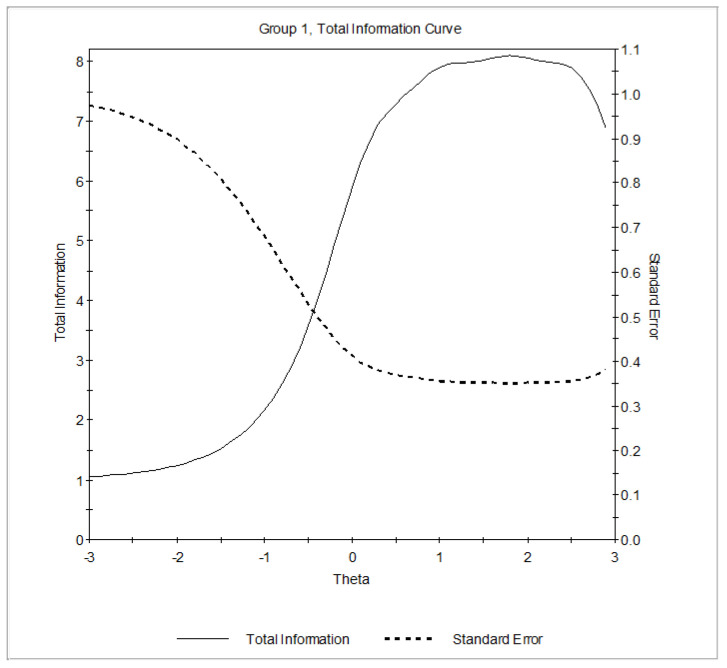
Test information function (TIF) of the BFAS under the graded response model (GRM). Latent trait (*θ*) is shown on the horizontal axis, and the amount of information and the standard error yielded by the test at any trait level are shown on the vertical axis.

Concerning reliability, the TIF indicated that the scale was sufficiently informative ranging from about −0.50 *SD*s below the mean to about +2.50 *SD*s above the mean. The amount of test information was ≥4, with values ≥7 starting from a mean level of the trait to +2.50. Since the associated reliability is 1 minus the inverse of the information the test provides (*r* = 1− [1/I]), we found that *r* was higher than 0.86 starting from this range of the trait (Figure 1). 

### 3.1. Validity 

As for validity, Pearson product–moment correlations for the BFSA using *θ values* attested that the relationships that were investigated were significant and in the expected directions (Table 2). Indeed, BFSA *θ score* was significantly and positively correlated with problematic social networking site use and Problematic Internet use. Additionally, it was significantly and positively correlated with loneliness and negatively with self-esteem. 

### 3.2. Gender Measurement Invariance

We measured gender DIF using the male group as the reference group and the female group as the focal group. Analyses were conducted using data from 1126 participants (Male = 558; Female = 568) because eight participants did not report their gender. Our results showed that the items did not show DIF (*p* values ranged from 0.05 to 0.96) (Table 3). After verifying gender invariance, we looked at gender differences by considering the BFSA *θ* score. Our results showed that there was no significant difference (*t*(1124) = −0.755, *p* = 0.451) between male (*M* = −0.02, *SD* = 0.85) and female participants (*M* = 0.018, *SD* = 0.89). 

### 3.3. Age Measurement Invariance 

We then analyzed age DIF, using the younger group as the reference group and the older group as the focal group. Analyses were conducted with 1124 participants because ten participants did not report their age. They were divided into groups by the median (21 years): Younger (522, <21 years) and older (602, ≥21 years). The items did not show DIF (*p* values ranged from 0.07 to 0.93), except Item 2 and Item 3 (Table 4). In particular, Item 2 reported significant DIF (*p* = 0.004) for the discrimination parameter, but not for the threshold parameters (*p* = 0.23). Item 3 reported a non-significant DIF (*p* = 0.45) for the discrimination parameter, but a significant one for the threshold parameters (*p* = 0.0001). 

Using all the other items as “anchor” items, the DIF detection procedure was then repeated. Anchor items were assumed to be without DIF and were used to estimate the trait and link the two groups in terms of trait levels. Anchor items are selected through a process of log-likelihood comparisons that are performed iteratively. During this iterative process, the status of item 2 changed in terms of the discriminative parameter (*χ*^2^(1) = 4.0, *p = 0*.05). However, the DIF status of Item 3 did not change in terms of the threshold parameter (*χ*^2^ (4) = 17.3, *p* = 0.002), thus indicating that the difference was in the same direction across the entire spectrum of the construct being measured for this item (i.e., one group was consistently more likely than the other to endorse an item at all levels of the trait). Nevertheless, since 17% of the BFAS items were non-invariant, we determined that the BFAS exhibited minor non-invariance as a whole. To understand the magnitude of the detected DIF for Item 3, effect size was calculated. It resulted negligible, as it was less than the cut value recommended by Raju [88].

After verifying age invariance, we looked at age differences by considering the *θ* scores at the BFAS. A significant difference was found between younger (*M* = −0.16, *SD* = 0.84) and older participants (*M* = 0.14, *SD* = 0.86), the latter of which had significantly higher levels of PFU (*t*(1122) = −5.92, *p* < 0.001, Cohen’s *d* = 0.35). 

### 3.4. Scoring System

Finally, looking at the TIF, we found that the highest amount of information ranged between 1.50 SDs and 2 SDs above the mean, i.e., from a trait level of 1.50 to a trait level of 2.00. The trait level ranging between 1.48 and 1.98 corresponded to a range in terms of summed score of ranged between 16 and 21, while the minimum cut-off scores are 12 or 18 and the maximum cut-off scores are 20 or 30 considering, respectively, the polythetic and the monothetic criterion. Thus, it seemed that the BFAS was more able to assess the range of the trait considered with the polythetic cut-off to determine PFU rather than the monothetic criterion. 

## 4. Discussion

Facebook use has become very popular over the past decade in all the world and, as a consequence, we assist to an increase of pathological use. For this reason, it is necessary to have sound measurement tools that can assess the problematic use. The present study investigated, for the first time, the psychometric properties of the BFAS among adolescents and young adults using an IRT approach. The IRT framework provides important advantages that can help to understand the potential of a given instrument.

Results demonstrated that the BFAS was unidimensional (e.g., [10,26,28]) and that the items were adequate in terms of their discriminative power and severity levels. In particular, items have an adequate differentiation in measuring the level of the trait around the mean to the high level of the trait, and the scale is sufficiently informative for these levels of the trait. Thus, with respect to previous psychometric studies on the BFAS, conducted through the Classical Theory of Test, the current IRT analysis allowed us to understand that this instrument is able to adequately measure both medium and high levels of PFU. 

Concerning measurement invariance, the BFAS resulted to be invariant across gender and age (as item 3 had a small and uniform DIF). In accordance with previous studies [10,33,34] we concluded that BFAS is gender invariant suggesting that it is equally useful for both men and women participants. Our results showed that there was no difference between men and women participants on the BFAS score. Previous results on gender differences were conflicting (e.g., [10,20,26,90] and a recent meta-analysis evidenced a small gender effect favoring female [6]. Gender differences have been explained by the evidence that women preferred social activities on the Internet (and this could put them at greater risk of developing Facebook addictive-like symptoms) (e.g., [10,15], whereas men are more attracted by leisure and gaming online activities [91]. However, Facebook provides both social and game applications and this aspect could at least in part be responsible for the non-emergence of differences on PFU levels between male and female participants in the current study. Inconsistent findings about gender differences in PFU require further research to investigate whether gender interacts with other variables (e.g., Facebook activities) in influencing PFU. As previously suggested [20], another aspect that should be considered in order to clarify gender differences issue is the interaction between gender and personalities traits on PFU. Xie and Karan [92] found that that the gender difference in PFU emerged only when trait anxiety was low.

Age invariance was tested for the Spanish version of the BFAS among a sample of university students in Peru [33], providing evidence for configural and metric invariance. In the present study, age invariance was obtained also at the scalar level, showing that Italian BFAS is invariant at a stronger level than the Spanish BFAS [33]. Our results showed that there was a significant difference between younger and older participants on the BFAS score, with older participants obtaining higher levels of PFU. This finding is consistent with Marino et al.’s [9] meta-analytic results on the association between PFU and psychological distress in adolescence and early adulthood. The above-mentioned meta-analysis showed that the association between PFU and psychological distress was stronger in samples with a higher mean age. As suggested by the authors, for younger people (i.e., the Digital Natives) using Facebook for most of the time in their everyday life could be perceived as “normative” and then less threatening for their psychological well-being.

We also examined the criterion validity of the BFAS by testing the relationships with theoretically related constructs and outcomes. As expected, BFAS scores were positively correlated with problematic Internet use and generalized problematic SNS use. The association between PFU and problematic Internet use had been previously reported (e.g., [27,28,29,30]), and could be explained by the fact that PFU could be considered a specific form of problematic Internet use [9]. Along the same lines, the relationship between PFU and problematic SNSs use has been previously reported e.g., [40,93], but the magnitude of the relationship found in the present study was not strong enough to conclude an overlap between the two constructs.

Finally, in accordance with previous studies [6], the higher the levels of PFU, the lower the levels of self-esteem and the higher the scores on the loneliness measure. However, longitudinal studies are needed in order to clarify the direction of these relationships, since low self-esteem and high loneliness could be predictors as well as negative consequences of PFU.

Concerning the scoring systems, we verified that the polythetic criterion for identifying Facebook addicts seems to be more suitable than the monothetic criterion as the proposed cut-off corresponds to a range of the trait in which the BFAS is highly informative. 

## 5. Strengths, Limitations and Further Research

The present study builds upon previous studies by adopting IRT analysis for the evaluation of the psychometric properties of the BFAS. The current findings provide further evidence of the satisfactory psychometric properties of the Italian BFAS among adolescents and young adults.

The BFAS items had adequate discriminative power in differentiating both medium and high levels of the trait. This means that BFAS is adequately informative for these levels of PFU. Thus, the instrument can be applied mostly for clinical purposes rather than for screening studies and preventative actions.

Moreover, a one-factor structure, invariant for age and gender, was found to be a good description of the data. Gender and age invariance of the BFAS is an important result as gender and age invariance instruments are required to better understand the ways in which these variables shape the experience of PFU, thus, to investigate differences and similarities in PFU between both men and women as well as younger and older individuals by conducting fair and unbiased comparisons.

As the authors of previous systematic reviews stated [94,95], future studies should propose hypotheses specific to different SNSs because not considering the type of sites included under the umbrella term of “social networking” might suppress relevant differences in people’s motivations to use SNSs [94,95], as well as in the negative consequences due to their excessive use, which may be different in relation to the specific type of SNS (e.g., [40,93]. Given its good psychometric properties, the use of the BFAS could be helpful for researchers to better understand the specificity of PFU in relation to other types of problematic SNSs use (e.g., Instagram, Snapchat, Twitter).

Based on these results, we suggest that the use of the Italian BFAS would be more helpful among the clinical population for diagnosing PFU and obtaining information about its severity rather than among the general population for screening Facebook addiction. However, future studies should be conducted on clinical sample by applying IRT in order to determine potential cut-off scores indicative of different levels of PFU severity. 

The present study has some limitations. Data were collected through non-probability sampling, which does not permit us to gather a representative sample of the whole population of Italian adolescents and young adults, thereby limiting the generalizability of the results. In particular, future studies should include university students from different majors which can contribute to the validity of the scale.

The cross-sectional design does not allow us to test the test-retest the reliability of the Italian BFAS (which has never been previously investigated) as well as its predictive validity. For this reason, longitudinal studies with larger and more representative samples are needed. 

## 6. Conclusions

Despite the limitations, this study offers evidence that the BFAS is a valuable and useful scale that can support future research on the pathologic use of Facebook. Indeed, when IRT analysis were used to assess its psychometric properties, the BFAS proved to be a unidimensional, gender and age invariant, and reliable measurement tool.

## Figures and Tables

**Table 1:** Mean (with standard deviation in brackets), Skewness, Kurtosis, standardized factor loadings (λ), item discrimination, and category threshold estimates (with the standard errors in brackets) of the items of the Bergen Facebook Addiction Scale (BFAS).

Item	M *(SD)*	Skewness	Kurtosis	*λ*	*a* *(SE)*	*b_1_* *(SE)*	*b_2_* *(SE)*	*b_3_* *(SE)*	*b_4_* *(SE)*
Spent a lot of time thinking about Facebook or planned use of Facebook	1.75(1.04)	1.4	1.4	0.72	1.78 (0.12)	0.21 (0.05)	1.17 (0.07)	2.02 (0.11)	2.62 (0.16)
Felt an urge to use Facebook more and more	1.69 (0.95)	1.3	1.1	0.87	3.05 (0.25)	0.22 (0.04)	1.05 (0.05)	1.79 (0.08)	2.57 (0.14)
Used Facebook in order to forget about personal problems	1.64 (0.99)	1.6	1.9	0.58	1.22 (0.10)	0.56 (0.07)	1.61 (0.11)	2.64 (0.19)	3.61 (0.28)
Tried to cut down on the use of Facebook without success	1.73 (1.03)	1.4	1.3	0.69	1.63 (0.12)	0.32 (0.05)	1.26 (0.08)	2.09 (0.12)	2.88 (0.18)
Become restless or troubled if you have been prohibited from using Facebook	1.41 (0.80)	2.2	4.9	0.80	2.26 (0.18)	0.81 (0.05)	1.63 (0.08)	2.30 (0.12)	2.97 (0.20)
Used Facebook so much that it has had a negative impact on your job/studies	1.53 (0.90)	1.8	2.7	0.63	1.37 (0.11)	0.71 (0.07)	1.71 (0.11)	2.71 (0.19)	3.75 (0.30)

Note. Standardized factor loadings λ are all significant at *p* = 0.001. Parameters were computed under the GRM model (*a* = discrimination, *b* = severity). *df* = degrees of freedom, *SE* = standard error.

**Table 2:** Descriptive statistics for the variables, and their correlations between the total scores at the BFSA *θ* scores.

	1	2	3	4	5
BFSA *θ* scores	-				
2. GPIUS2 summed scores	0.43 ***	-			
3. IAT summed scores	0.48 ***	0.69 ***	-		
4. ILS summed scores	0.15 *	0.43 ***	0.35 ***	-	
5. RSES summed scores	−0.18 *	−0.28 ***	−0.29 ***	−0.49 ***	-
*M*	0.00	26.61	41.84	33.07	27.15
*SD*	0.87	15.03	11.34	6.78	5.36

Note. BFSA = Bergen Facebook Addiction Scale; GPIUS2 = Generalized Problematic Internet Use Scale 2; IAT = Internet Addiction Test; ILS = Italian Loneliness Scale; RSES = Rosenberg Self-Esteem Scale; * *p* < 0.05, *** *p* < 0.001.

**Table 3:** Differential item functioning (DIF) of discrimination and severity parameters across gender.

		*a*DIF			*b*DIF	
Item	*χ* ^2^	*df*	*p*	*χ* ^2^	*df*	*p*
1	0.00	1	0.87	0.20	4	0.99
2	0.40	1	0.54	1.3	4	0.86
3	0.20	1	0.67	9.6	4	0.05
4	0.50	1	0.46	1.4	4	0.85
5	0.40	1	0.51	1.3	4	0.86
6	4.3	1	0.04	6.6	4	0.16

Note. DIF = Differential Item Functioning, a = discrimination, b = severity, df = degrees of freedom, χ^2^ = chi-square value, *p* = probability value.

**Table 4:** Differential item functioning (DIF) of discrimination and severity parameters across age.

		*a*DIF			*b*DIF	
Item	*χ* ^2^	*df*	*p*	*χ* ^2^	*df*	*p*
1	2.4	1	0.12	5.4	4	0.25
2	8.1	1	0.004	5.7	4	0.23
3	0.60	1	0.45	22.8	4	0.0001
4	2.3	1	0.13	5.7	4	0.23
5	0.20	1	0.68	3.8	4	0.44
6	0.00	1	0.93	8.6	4	0.07

Note. DIF = Differential Item Functioning, a = discrimination, b = severity, df = degrees of freedom, χ^2^ = chi-square value, *p* = probability value.

## Data Availability

The data presented in this study are available on request from the corresponding author. The data are not publicly available due to privacy issues.
